# Environmental enrichments and data-driven welfare indicators for sheltered dogs using telemetric physiological measures and signal processing

**DOI:** 10.1038/s41598-024-53932-1

**Published:** 2024-02-09

**Authors:** Tiziano Travain, Teddy Lazebnik, Anna Zamansky, Simona Cafazzo, Paola Valsecchi, Eugenia Natoli

**Affiliations:** 1 Department of Chemistry, Life Sciences, and Environmental Sustainability, University of Parma, Parma, Italy; 2 Department of Mathematics, Ariel University, Ariel, Israel; 3 Department of Cancer Biology, Cancer Institute, University College London, London, UK; 4 Information Systems Department, University of Haifa, Haifa, Israel; 5 Milan, Italy; 6 Sleep Disorders Center, Department of Medicine and Surgery, University of Parma, Parma, Italy; 7 Canile Sovrazonale, ASL Roma 3 (Local Health Unit Rome 3), Rome, Italy

**Keywords:** Scientific data, Animal behaviour, Animal physiology

## Abstract

Shelters are stressful environments for domestic dogs which are known to negatively impact their welfare. The introduction of outside stimuli for dogs in this environment can improve their welfare and life conditions. However, our current understanding of the influence of different stimuli on shelter dogs’ welfare is limited and the data is still insufficient to draw conclusions. In this study, we collected 28 days (four weeks) of telemetry data from eight male dogs housed in an Italian shelter for a long period of time. During this period, three types of enrichment were introduced into the dogs’ pens for one week each: entertaining objects, intraspecific, and interspecific social enrichment, by means of the presence of female conspecifics and the presence of a human. To quantify their impact, we introduce novel metrics as indicators of sheltered dogs’ welfare based on telemetry data: the variation of heart rate, muscle activity, and body temperature from an average baseline day, quality of sleep, and the regularity for cyclicity of the aforementioned parameters, based on the day-night cycle. Using these metrics, we show that while all three stimuli statistically improve the dogs’ welfare, the variance between individual dogs is large. Moreover, our findings indicate that the presence of female conspecific is the best stimulus among the three explored options which improves both the quality of sleep and the parameters’ cyclicity. Our results are consistent with previous research findings while providing novel data-driven welfare indicators that promote objectivity. Thus, this research provides some useful guidelines for managing shelters and improving dogs’ welfare.

## Introduction

Since 1991, in Italy the euthanasia of unowned free-ranging dogs and cats has been illegal, except in cases of proven dangerousness or incurable diseases (national law n. 281/1991 “Legge quadro in materia di animali di affezione e prevenzione del randagismo”, en: “Framework law regarding pet animals and stray dogs’ prevention”). Among other consequences, this law raised the issue of ensuring an adequate level of welfare for sheltered dogs since the adoption rate is lower than the entrance rate, and consequently, many dogs will spend a significant part of their lives (sometimes their entire life) into shelters^1^.

Nevertheless, welfare is a theoretical and elusive concept and its measurement and precise definition are still widely debated^2,3^. It concerns the physical and mental health of animals, since poor life conditions influence animal behavior and physiology and could cause pre-pathological or pathological conditions. Ensuring animal welfare is not an easy task and for many years much emphasis has been placed on measuring the resources provided to animals in terms of space, food, water, and shelter (resource-based approach). In the last years, researchers shifted their attention to specie-specific behavioral needs, physical and mental health to measure the quality of life of a variety of domestic animals (animal-based approach^4^) and to develop different measurements of welfare and hazards assessment using behavioral and physiological indicators^5–10^.

In Italy, requirements for housing dogs in shelters are regulated by national and regional guidelines that define resources that should be offered to dogs (space provided for each dog, floor type, bedding, food availability) but do not give stringent indications on their management (social vs individual housing, walking, playing, training and socialization with humans) and the consequence is a high variability in management strategies across Italy: it is still possible that dogs are individually housed in paved pens without access to a grass area to exercise and to socialize with either conspecific or humans^11^. Poor management together with the unavoidable exposure to the unpredictable/uncontrollable shelter environment could lead dogs to develop stereotypes and stress-related behaviors^11–16^.

Over the past years, to improve dogs’ welfare and to mitigate the detrimental effect of sheltering, environmental enrichments (EE) have become commonly used^17^. Two main types of EE have been tested in dog shelters: animate, i.e. social contact and/or activity with conspecifics and humans, and inanimate, for example toys, different bedding and food, auditory and olfactory stimulation^9,11^. Social contact, with either conspecifics or humans, is one of the most studied EE for sheltered dogs. Hubrecht and colleagues^18^ and Mertens and Unshelm^19^ compared behavioral patterns of solitary and group-housed dogs living in animal shelters and laboratories, and their results highlighted how solitary dogs were more inactive and expressed more stereotypical behaviors rather than group-housed dogs, with the latter expressing more exploratory behaviors, likely because of the increased olfactory stimulation. Willen and colleagues^20^ showed that sheltered dogs experiencing 15 min of quiet, positive human interaction had a reduced plasma cortisol response to stress. Other studies obtained similar results, confirming the positive effect of interaction with humans also for fearful dogs^14,20–22^ while showing that none or irregular activity with humans and other dogs can be detrimental and can promote the onset of behavioral problems^7^. Most used inanimate enrichments are toys^23^, pen furniture^24^, music or audiobooks^25,26^, and olfactory stimuli^17^, or all these items together^11^. Even if the obvious aim of providing enrichment is to enhance the quality of life of the dogs, toys’ effectiveness is somehow limited. Dogs exposed to different toys spent little time playing and their interest waned over time^27^. A slightly higher effect is achieved when the toys are actually used by a person: dogs are more willing to friendly approach the experimenter^28^.

Although dozens of studies have examined the effects of specific environmental enrichments on the behavior and physiology of sheltered dogs, a consensus on which are the best measures to estimate the effect/value of these enrichment procedures has not been achieved yet because results are sometimes difficult to interpret in term of welfare and differ from one study to another. Furthermore, at the best of our knowledge, no studies assessed the prolonged effects on behavior and physiology of a complex enrichment program that involves both animate and inanimate forms of enrichment. The vast majority of existing studies focus on a single stimulus or a set of similar stimuli and dogs are monitored for a very limited time period, ranging from the first minutes to a few days^9^.

The present study monitors physiological parameters with a telemetry system. Currently, telemetry systems can collect blood pressure and flow, heart rate, electrocardiogram, electroencephalogram, electromyogram, respiratory rate, pH, body temperature, and activity indexes^29^. In biomedical research, they have several advantages: (1) reduction of distress compared with conventional measurement techniques, allowing monitoring of physiological parameters in conscious, freely moving laboratory animals; (2) reduction of animal use^30^; (3) unrestricted continuous data collection for prolonged periods of time without the need of any special animal care; (4) availability for use in a wide range of laboratory species (e.g. mice, monkeys, some fishes). Some disadvantages of telemetry should be also considered: (1) not negligible cost; (2) implantation surgery of the transmitter. The aim was to assess physiological responses of shelter dogs to 3 different environmental enrichments across a 4-week period. After one week of recording dogs under routine management conditions (baseline), they were exposed to three different types of environmental enrichment interventions, a different one for every week. Physiological data was then analyzed with an unprecedented data-driven approach that is able to cope with the very large amount of data generated by telemetry to propose new welfare indicators. More specifically, we elaborate and propose three ‘welfare metrics’ that can be extracted from telemetry data using signal processing techniques: 1. Predictability; 2. sleep quality; and 3. day-night cycle.

Predictability is a metric that allows measurement of the similarity of a given day to an artificial ‘average day’, calculated from an individual’s physiological parameters (HR, T, and muscular activity) during the baseline week. In a controlled and stable environment, whether fit or unfit, animals reach their homeostasis level, which is constant. For example, it has been shown that in laboratory rats, any new stimulus, e.g. a change in lighting, sudden loud noises, a change in diet, or even cleaning procedures, caused a modification in individuals’ homeostatic equilibrium^31^. In this study we predict the EE changes the calculated value of the artificial 'average day' of dogs, because any modification in a static environment modifies the way the animal perceives it, causing a homeostatic disequilibrium.

Sleep quality measures how long dogs sleep at night and how many awakenings they show during sleep. Despite being poorly studied in shelter dogs^32^, sleep quality is an important parameter to assess welfare because it is well-known that sleep is a fundamental part of an individual homeostatic process^33^. Furthermore, sleep structure is associated with environmental factors and there is a reciprocal influence between sleep and activities performed when awake^34,35^. In dogs, Schork and colleagues^36^ explored the behavior and sleep habits of 13 laboratory dogs housed in outdoor pens with natural light and ambient temperature. Dogs slept mainly during the night, considering both sleeping time and bouts. A higher number of sleeping bouts, which define a very fragmented sleep, caused an increase in time spent asleep during the night and increased inactivity and maintenance behaviors during the following day. We hypothesize that better sleep quality is associated with a lower number of sleeping bouts and a shorter duration of awakenings from sleep at night, and with longer sleep intervals between each awakening.

Day-night cycle measures the difference between days and nights in dogs’ heart rate and muscular activity: some experimental evidence suggests that a strong difference between daily and nocturnal activities may be a valid parameter to monitor the adaptability of dogs to a shelter environment. It has been shown that dogs in shelters exhibited higher nocturnal activity in the first two days after intake, than after a 12-day habituation period^37^. Maintaining an appropriate day and night activity cycle is important because a disrupted circadian rhythm can cause both mental and physical problems^38^. Since sleep deprivation and disruption of circadian rhythms can lead to various problems such as locomotor patterns and general activity modifications, increased aggressive behavior, anxiety-like behaviors, and decreased cognitive, and health issues^36,38^, we hypothesize that a larger difference between diurnal and nocturnal activity is associated with better welfare.

We further employ the three metrics to study the individual patterns of each dog and their responses to the various stimuli. Finally, we also investigate the correlation between the different physiological parameters (temperature, heart rate, and muscular activity).

## Results

First, we evaluated the predictability metric (p), namely how predictable dog’s physiological parameters are when compared to an “average” baseline day, for each dog for each week. Mean and standard deviation of each week are reported in Table 1. For all dogs, the three weeks with environmental enrichments (EE), compared to the baseline (BL), resulted in lower values of predictability. Moreover, the human presence (HP) and the female dog (FD) resulted in similar levels of predictability which both were lower compared to the objects (OB). A non-parametric k-sample Anderson–Darling test revealed that all EE were significantly different from the BL ($p=0.031$) and that both HP and FD were statistically significantly different from OB ($\mathrm{OB vs HP}: p=0.043, \mathrm{OB vs FD}: p=0.049$) while HP and FD are not ($p=0.246$).

**Table 1:** Mean and standard deviation of the predictability metric ($p$) for each dog for each week (Baseline: BL; Objects: OB; Human presence: HP: Female dog: FD).

Dog	Baseline BL	Objects OB	Human presence HP	Female dog FD
Brad	0.85	0.58	0.46	0.50
Buck	0.78	0.62	0.52	0.56
Jack	0.91	0.70	0.63	0.58
Brando	0.80	0.59	0.55	0.59
Lenticchia	0.84	0.66	0.63	0.57
Vin	0.93	0.79	0.81	0.74
Sparrow	0.87	0.70	0.71	0.68
Scotty	0.90	0.68	0.58	0.61
**Mean ± STD**	**0.860 ± 0.049**	**0.665 ± 0.064**	**0.611 ± 0.103**	**0.603 ± 0.069**

Values are unitless in the [0,1] interval.

In order to evaluate the sleep quality, one was first required to set the values of ${\zeta }_{1}, {\zeta }_{2},$ and ${\zeta }_{3}$. For the purpose of making all three components of the sleep quality metric identical, for each dog, we first computed the largest value of ${s}_{i}, {s}_{n},$ and ${s}_{\tau }$ indicated by ${s}_{i}*, {s}_{n}*,$ and ${s}_{\tau }*$, respectively, and setting the values of ${\zeta }_{1}, {\zeta }_{2},$ and ${\zeta }_{3}$ to be $\frac{1}{3{s}_{i}*} , \frac{1}{3{s}_{n}*},$ and $\frac{1}{3{s}_{\tau }*}$, respectively. Using these values, it was possible to compare the influence of the different EE on the dogs’ population sleep quality but did not allow comparison between dogs. Moreover, to make the metric values easier to analyze, we linearly transferred its values between $[0, 1]$ rather than $[\frac{-1}{3}, \frac{2}{3}]$ by adding $\frac{1}{3}$ to all results. The mean and standard deviation of each week are reported in Table 2. The OB did not have much effect on the results. On the other hand, both the HP and FD showed an increase in sleep quality while also increasing the differences between the dogs, as indicated by the standard deviation. Statistically, the non-parametric k-sample Anderson–Darling test revealed that only the FD was statistically significantly different from the baseline ($p =0.030$) while the other cases were not statistically significantly different from each other ($p=0.096$).

**Table 2:** Mean and standard deviation of the sleep quality ($s$) for each dog for each week (Baseline: BL; Objects: OB; Human presence: HP; Female dog: FD).

Dog	Baseline BL	Objects OB	Human presence HP	Female dog FD
Brad	0.27	0.28	0.32	0.40
Buck	0.22	0.19	0.16	0.25
Jack	0.38	0.41	0.40	0.48
Brando	0.35	0.35	0.38	0.42
Lenticchia	0.28	0.26	0.23	0.31
Vin	0.31	0.34	0.39	0.39
Sparrow	0.34	0.32	0.36	0.38
Scotty	0.35	0.32	0.39	0.45
**Mean ± STD**	**0.312 ± 0.049**	**0.309 ± 0.062**	**0.329 ± 0.083**	**0.385 ± 0.069**

Values are unitless in the [0,1] interval.

In a similar manner, the results for the day/night cycle metric ($c$) are presented in Table 3. Both the HP and FD caused a significant increase in the* c* metric while the OB caused only a slight increase. The non-parametric k-sample Anderson–Darling test revealed that both the HP and FD were statistically significantly different from the BL ($\mathrm{BL vs HP}: p = 0.039,\mathrm{ BL vs FD}: p = 0.046$) while the OB is not ($p=0.159$). Moreover, HP and FD showed a significantly higher day/night metric ($c$) than the OB ($p = 0.044$) but their values were not significantly different from each other ($p= 0.202$).

**Table 3:** Mean and standard deviation of the cyclicity metric ($c$) for each dog for each stimulus type. (Baseline: BL, Objects: OB, Human presence: HP, Female dog: FD).

Dog	Baseline BL	Objects OB	Human presence HP	Female dog FD
Brad	0.56	0.58	0.65	0.64
Buck	0.51	0.49	0.52	0.50
Jack	0.59	0.61	0.63	0.67
Brando	0.60	0.65	0.64	0.63
Lenticchia	0.64	0.60	0.69	0.63
Vin	0.49	0.56	0.62	0.59
Sparrow	0.56	0.53	0.64	0.60
Scotty	0.57	0.58	0.53	0.62
**Mean ± STD**	**0.565 ± 0.045**	**0.580 ± 0.054**	**0.615± 0.055**	**0.610 ± 0.047**

Values are unitless in the [0,1] interval.

As the results above were obtained using all three physiological parameters, it was of interest to examine if the same tendency of the presented outcomes over the dogs’ population was kept if only a subset of these parameters was available. In other words, checking the “consistency” among the subsets of the three physiological parameters. To measure this “consistency”, or “similarity” among the parameters, we used the linear coefficient fitting values, obtained on the average data for each parameter subset we considered. The muscular activity was included in all subsets as it was required by definition for the sleep quality metric.

Table 4 provides a summary of this analysis where the rows are the different subsets of physiological parameters, presenting their similarity in the form of the coefficient of a linear fitting of the mean result for each subset. Notably, the sleep quality metric was identical for all the considered subsets as it was defined only by the activity parameter. The similarity of the coefficients among the different considered subset implies that they were consistent in the sense of showing similar trends.

**Table 4:** Similarity of the proposed metrics with different physiological parameter configurations.

Telemetry subset	Predictability (*p*)	Sleep quality (*s*)	Cyclicity (*c*)
Activity	− 0.035	0.023	0.011
Activity and temperature	− 0.040	0.023	0.009
Activity and heart rate	− 0.062	0.023	0.019
All parameters	− 0.084	0.023	0.017

The values presented in the table are the linear coefficient fitting values, obtained on the average data for each parameter subset. Values are unitless in the [− 1,1] interval.

## Discussion

In this study we explore the use of telemetry and signal processing to pioneer data-driven precise and objective welfare indicators for sheltered dogs exposed to various enrichment stimuli. After a baseline week, dogs were exposed to three one-week long different environmental enrichments: a non-social one and two social ones. During the experiment, dogs’ heart rate, core temperature, and muscular activity were recorded every minute and data were used to develop three different metrics, that are predictability, how similar daily dogs’ physiological parameters are to an “average” day with their normal routine, day/night cycle, which measures the average difference between the day and night phases in the dog’s activity, and sleep quality that considers the number of times dogs awake from sleep at night, the mean interval between two awakenings, and the mean length of each awakening. The value of this approach lies in the huge amount of data collected objectively, without the possibility of influencing the physiological parameters with survey methods involving the manipulation of the dogs during data collection.

The results of predictability show that any change in the standard environment in which dogs live lowers the predictability of the analyzed parameters. Intuitively, even if these results are consistent with the literature findings in different animals, such as calves^39^ or rats^31^, this metric alone does not allow to claim that these changes are improving dogs’ welfare. This is due to the fact that, although significant, the metric does not give a 'qualitative' difference in positive or negative direction. It only stated that the parameters changed during the weeks with EE introductions meaning that the environmental changes were perceived by the individuals as something impactful, breaking the existing homeostatic equilibrium and driving them to a novel one^31^. However, the results of the sleep quality and cyclicity analysis, shown in Tables 2 and 3, confirmed our hypothesis: both were positively influenced by the social stimuli compared to the week in which enrichment was not present, and the dogs showed better sleep quality and a regular difference between the frequencies of the daytime and night-time metrics, whereas environmental enrichment with the objects did not produce statistically significant changes. These results confirm what found by Wells^27^, i.e. different types of objects, although pleasant for dogs, are not effective environmental enrichments per se and, on the other hand, that socialization with both humans and conspecifics is certainly destabilizing but usually has positive effects^40–42^.

Notably, the presence of the female stands out as the most effective stimulus, positively impacting both sleep quality and cyclicity. This is consistent with the existing literature regarding social environmental enrichments: dogs need a physically^13,24^ and socially^11,17^ stimulating environment in order to avoid stress-related behavior, such as displacing activities and stereotypies^11,43^, and abnormal HPA axis activation^44^ while promoting natural behavior^45,46^. It is noteworthy that the improvement in sleep quality and cyclicity in the presence of the conspecific female occurred in a shelter, i.e. an environment where one of the main causes of sleep disturbance in dogs is noise pollution from the barking of other dogs^47^. This is a very interesting result, especially when compared with the results of the study by van der Laan and colleagues^48^ in which moving to a socially stimulating environment, such as a new home, improves significantly sleep quality in dogs in terms of both prolonged bouts of sleep and reduced nocturnal activity and that the sleep quality improves over time. Moreover, one indirect support to our results might come from the results of Carreiro and colleagues^49^ who found that the sleep EEG characteristics of owned dogs improve when, even if in a novel environment, they sleep with the owner, as the latter might function as a safe haven. It is possible that also a conspecific has a positive impact on sleep quality of these dogs although safe haven effect between cohabitating dogs has not been yet demonstrated.

Even the human presence stands as an effective stimulus, despite being discontinuous as the female volunteer was present daily only for 2-h periods. Considering dogs’ need for a social environment this is a good but not obvious result, since in the literature there is contrasting evidence regarding the usefulness of a limited human contact in sheltered dogs. In a shelter setup similar to the present one, dogs living alone in their pen and receiving regular daily visits by the same person that stood outside the pen (in the present experiment the volunteer was inside), did not diminish the frequency of displacement behaviors and oral stereotypies and increased concentration of fecal cortisol metabolites^11^. On the other hand, positive effects of interactions with a human and negative effects of its absence or irregularity have been reported by different authors. Bergamasco and colleagues^40^ reported that a long term (3 days per week, for 8 weeks) positive reinforcement based training had a positive effect on behavior and could affect welfare physiological indicators. Menor-Campos and colleagues^21^ found that short (25-min long) but repeated dog–human interactions diminished dogs’ stress response (cortisol level) and improved their behavior. Arena and colleagues^7^ showed a relationship between the lack of exercise in outdoor fenced areas with insurgency of aggressive behaviors.

Both socialization with humans and conspecifics and good sleep quality can improve dogs’ desirability and facilitate adoption. Singly housed dogs spend most of their time at the back of the pen, which is considered undesirable by adopters^17^, while socialization reduces separation-related anxiety problems after adoption^50^ and human contact increases the time spent at the front of the pen^51^. On the other hand, a bad quality of sleep negative affects physical and mental health, causes the dogs to be bad-tempered and/or sleepy, and reduces activity time during daytime^36,38^.

Interestingly, the analysis of present results cannot ignore that there was a significant variation in the individual responses of the dogs to these stimuli, as it often occurs in shelter dogs^11,52^. While toys have elicited mild reactions in all dogs and therefore it is difficult to say if this situation had been perceived as positive or negative, social enrichments elicited strong and very different reactions among dogs. This can be due to several factors: firstly, as often happens with shelter dogs, nothing was known on their life before entering the shelter; secondly, dog personality was not assessed for the subjects of the study and it is now well known that different individuals show different behavioral syndromes when have to cope with a stressful environment^52^. Therefore, it is possible that past experiences and personality differences played an important role in modulating the dogs’ responses to enrichments. Thus, although the overall results on social stimuli encourage their use, it is also important to consider that there is not a single path to improve sheltered dogs’ welfare and that the enrichments should be proposed after a careful evaluation on a case-by-case basis.

This study features a limited number of dogs, that are all males and have an unknown background. A larger dog population including dogs of different age, size, sex, intact or neutered could reveal more interesting connections in the data. Following these limitations, future studies could expand the sample size and investigate a wider range of enrichment options. Although it is very difficult obtaining a detailed picture of previous experiences of sheltered dogs, it will be a paramount issue having an accurate as possible analysis of dogs’ background since it has been showed in the study of van der Laan and colleagues^48^ that the presence or absence of a shelter history and being a stray or a relinquished dog affected in various way the quality of sleep of dogs both in shelter and post-adoption. Moreover, as revealed by Table 4, one can use as little as only an activity parameter to statistically obtain the same results as all three parameters.

The importance of behavioral observations should not be underestimated, as it is an immediate and obvious approach to assess ongoing stress or health issues, therefore the behavioral data collected during this study will be analyzed using computer vision techniques, due to the enormous quantity of data that makes manual coding an impracticable way, and published in a following article. Long-term monitoring of dogs in different shelter environments could provide valuable insights into the sustained effects of enrichment. Additionally, exploring the potential benefits of combining multiple enrichment strategies could help refine welfare management practices in shelters and it could be beneficial to examine the correlation between the proposed welfare metrics with the dog’s clinical state, as it is known that the two are closely connected^53,54^.

## Methods

### Ethics statement

The study was approved by the Italian Ministry of Health (permission No. D6SA/VI/7992-P, 19th July 2007). All animals were cared in accordance with current Italian laws regarding animal welfare and stray dogs’ prevention. Experimental procedures followed the ARRIVE guidelines.

### Animals and housing

The study was conducted at the private dog shelter “Centro Cinofilo del Lago” in Bracciano (Rome, Italy). Pens were arranged on a single row, all facing a hallway. Each pen had indoor and outdoor portions measuring 1.5 × 2.0 m each, and an opening in the wall allowed dogs free indoor/outdoor access. Adjacent pens were separated by a 1-m solid wall, on which is fixed a metal mesh reaching the ceiling. This allows dogs to stand up to look into other pens. The front and the rear sides have only wire mesh. The door is located in the front, inside, towards the hallway. The outside part of the pen overlooks the countryside.

The subjects were eight intact healthy mixed-breed (1 German shepherd mix, 1 Rottweiler mix, 1 hunting-type mongrel, 1 shepherd-type mongrel, 4 mongrels) male dogs, with an estimated age lower than five years. The sample included six medium-sized dogs and two large-sized dogs, all singly housed; their permanence in the shelter ranged between three months to two years when the study started. The subjects were intact because the neutering and the postoperative consequences could have altered the data collection. In order to minimize stress for the animals, the neutering was performed contextually to the removal of the telemetry system.

The health status of the dogs was checked on a regular basis by a veterinarian. Due to management rules, dogs were never taken out of the pen for a walk, except for rare displacements due to the reorganization of shelter or medical care, but this did not happen during the study. Dogs were exposed to human contact once per day during routine husbandry (feeding and cleaning); no additional interaction or socialization opportunity was provided on a regular basis.

## Experiment’s settings

### Data collection

Dataquest A.R.T. CTA-D70 system telemeters (Data Sciences International, MN, USA), consisted of a disk (diameter: 5 cm, thickness: 0.8 cm) from which two electrodes departed. These were two copper wires covered by rubber of 20 cm, the ends were placed one under the axillary cavity, the other near the heart. The use of the device involves its implantation under the skin in a ventral position, immediately below the sternum. For this purpose, the device is made of biocompatible material and packaged in a casing that preserves sterility. The implantation was performed during a surgical procedure in which the dog was anesthetized with an intramuscular injection of medetomidine hydrochloride (Domitor 1 mg/ml; 10 mg/kg; Vétoquinol Italia S.R.L.., Italy) and propofol (Diprivan 20 mg/ml; 2–4 mg/kg; Tuttofarma S.R.L., Italy).

At the end of the test period, the removal of the device was carried out in conjunction with the neutering surgery.

Collected data were transmitted via radio to receivers placed inside the pen. Receivers were antennas contained inside metallic plates. In each pen, there were four receivers, two inside and two outside, two on the right side and two on the left side; all of them were mounted on the wall about 50 cm above the ground. From the receivers, a cable departed to reach the Data Exchange Matrix connected to a personal computer. Cables were channeled in a plastic tube exiting the shed and reaching a nearby workstation about 5–6 m away. The Data Exchange Matrix had the following functions: (1) energizing the receivers; (2) converting and sending to the PC the collected data; (3) calculating the muscular activity index.

The physiological parameters collected were body temperature (°C), heart rate (bpm), and muscular activity (measured in movement units). As previously said, the latter is calculated by the Data Exchange Matrix: every time dogs moved, the signal emitted by the transmitter to the receivers varied in strength, orientation, and distance from the antennas allowing the software to generate the movement units. The sampling rate allowed one reading per minute. These values were the mean values of the minute itself. Data were collected every day, 24 h/day, and they formed a time series. Considering one reading per minute, during the whole experimental period we obtained 60 readings per hour, 1,440 readings per day, 10,080 per week; this means that we collected 40,320 readings per dog for a total of 322,560 readings. This amount of data offers a unique possibility to develop and test metrics using a data-driven approach in order to obtain objective parameters to measure the effect/values of EE and welfare in sheltered dogs.

### Experiment procedure

After implantation of the telemeter, the aforementioned physiological parameters were recorded for a period of two weeks in order to monitor the post-operative course and to exclude the occurrence of inflammation and/or infection with temperature elevation. This did not occur in any of the eight dogs studied. The data collected during the 14 days following surgery were then discarded. At the end of this post-operative period, the actual study began. In the first week, all dogs were in the same standard housing condition (baseline). After baseline, each enrichment was proposed for one week and the order of presentation varied among dogs according to the scheme reported in Table 5. Due to the limited number of subjects and external constraints, a complete randomization of EE order presentation was not possible.

1. Baseline (BL): Standard housing condition, the dog was alone in the pen without any kind of enrichment; in the pen, there was only a wooden platform to sleep, a metal bowl for the food, and one for the water.
2. Objects (OB): enrichment was provided in the pen in the form of a cot, a perforated rubber ball containing kibble, a rubber ball, a rubber chewing toy, a knotted rag soaked in urine from a female dog unknown to the test subject, and a natural beef bone. In mid-week, the enrichment was renewed by bringing in more biscuits and again impregnating the rag with the smell of a female.
3. Human Presence (HP): once a day, after the morning husbandry chores, one female volunteer, familiar to the dogs, interacted with each dog for two hours (playing, cuddling, or just keeping the dog company if it did not want to physically interact) inside their home pen; the same person visited the same dog for the duration of the HP week.
4. Female Dog (FD): the dog shared its own home pen with an unknown, spayed female; a second wooden platform and a second food bowl were added. A few days before introducing the female into the pen, dogs were tested for compatibility, as it is normal practice in shelters when choosing two partners to cohabit in the same space. During the study, neither aggression nor bites were recorded between the partners matched for the purposes of this study.

**Table 5:** Schedule of presentation of environmental enrichments for the eight dogs.

Dog	1st week	2nd week	3rd week	4th week
Lenticchia	BL	OB	FD	HP
Jack	BL	OB	FD	HP
Buck	BL	OB	FD	HP
Vin	BL	HP	OB	FD
Brando	BL	FD	HP	OB
Scotty	BL	FD	OB	HP
Brad	BL	FD	OB	HP
Sparrow	BL	FD	OB	HP

BL = baseline week; OB = week with objects; HP = week with human presence; FD = week with female dog presence.

### Telemetry-Based welfare metrics

We define three welfare metrics based on telemetry data: predictability, day/night cycle, and sleep quality. The **predictability metric**, *p*, captures how predictable dog’s physiological parameters are, or in other words how similar the dog’s days are to an *“average”* day. The **day/night cycle metrics**, *c*, measures the average difference between the day and night phases in the dog’s activity. The **sleep quality metrics** measures (i) ${S}_{n}$—the number of times dogs awake from sleep at night, (ii) ${S}_{i}$—the mean interval between two awakenings, and (iii) ${S}_{\tau }$—the mean length of each awakening. The metrics return degree values in the interval [0,1].

For formalizing our suggested welfare indicators, we note that the 24 h day is divided into two parts: diurnal and nocturnal according to daylight times (Table 6). Furthermore, the dog’s state at time point t is represented by three signals heart rate ($h(t)\in {R}^{+}$), core temperature $(\tau (t)\in {R}^{+})$, and activity *(*$a(t) \in {R}^{+}$*).*

**Table 6:** Mean starting and ending times of daylight, rounded to the nearest minute, during the four weeks.

Dog	1st week	2nd week	3rd week	4th week
Lenticchia	05:35–19:07	05:22–19:15	05:10–19:23	04:59–19:31
Jack	06:13–17:42	06:21–17:39	06:27–17:38	06:33–17:39
Buck	05:00–21:13	04:53–21:21	04:45–21:28	04:40–21:34
Vin	06:00–17:51	06:09–17:46	06:18–17:41	06:25–17:39
Brando	05:53–17:58	06:09–17:46	06:17–17:42	06:24–17:40
Scotty	04:37–21:39	04:34–21:44	04:34–21:48	04:35–21:50
Brad	04:37–21:40	04:34–21:45	04:34–21:49	04:35–21:51
Sparrow	04:37–21:40	04:34–21:45	04:34–21:49	04:35–21:51

For the analysis, daily values were used.

Let us assume all metrics receive as an input a matrix, $I\in {R}^{4\times n}$*,* where $n \in N$ is the number of measurements and each measurement (i.e., row) in the data is represented by the heart rate ($h\in {R}^{+}$), core temperature $(\tau \in {R}^{+})$, activity *(*$a\in {R}^{+}$*)*, and a binary part of day indicator (diurnal/nocturnal)*.* Similarly, $b\in N$, is the number of measurements for the *baseline* period in the dataset. Hence, the three metrics take the following forms:

$$p(I) :=\frac{1}{3}{\sum }_{i=1}^{3}\left(\sqrt{\frac{{\sum }_{j=1}^{b}({\mu }_{i}-{I}_{i,j})}{b}}\right)$$

$$s(I) :=\frac{1}{D}{\sum }_{i=1}^{D}{({\zeta }_{1}{s}_{i} -\zeta }_{2}{s}_{n}-{\zeta }_{3}{s}_{\tau }),$$

$$c(I) :=\frac{1}{D}{\sum }_{i=1}^{D}\left(\frac{1}{3}{\sum }_{i=1}^{3}\left(\frac{{\mu }_{i}({{k}_{d}}^{i})-{\mu }_{i}({{k}_{n}}^{i})}{max({\mu }_{i}({{k}_{d}}^{i}), {\mu }_{i}({{k}_{n}}^{i}))}\right)\right)$$

Such that ${\mu }_{i}:=\frac{1}{n}{\sum }_{j=1}^{n}({I}_{i.j})$, where $D\in N$ indicates the number of days in the dataset (computed by the number of sequential pairs of diurnal and nocturnal indicators, ${j\in [\mathrm{1,2},3]: \zeta }_{j}\in {R}^{+}$ are the weights of the three properties of sleep quality. ${s}_{n}$ is a function that shows how many times a dog wakes up at night by computing the mean and standard deviation of the activity metric on the entire dataset, and computing a passing of the mean plus one standard deviation during night time. ${s}_{i}$ computed the mean interval between two awakenings. ${s}_{\tau }$ computed the mean duration of awakenings. ${{k}_{d}}^{i}$ and ${{k}_{j}}^{i}$ are the set of diurnal and nocturnal samples, respectively, of the ${i}_{th}$ day.

In addition, in order to capture the average behavior of the proposed metrics for different configurations of available data, we computed the linear regression coefficient on the mean result of each subset of the metrics.

### Data analysis

We computed the three metrics for each dog and each week (baseline, objects, human presence, and female dog) separately.

Afterward, since data is not normally distributed, we used the non-parametric k-sample Anderson–Darling with post-hoc t-tests and Bonferroni correction to compare the metrics between weeks for each dog separately. Mean ± STD were also calculated as aggregate measure across all dogs.

The p-value for statistical significance was set at $p\le 0.05$. All analyses have been conducted using the Python programming language (version 3.8.2).

## Data Availability

The raw data and analysis of this study are available from the corresponding author on request.
